# The moderator effect of sex on attitude toward communication, emotional intelligence, and empathy in the nursing field

**DOI:** 10.1590/1518-8345.2018.2969

**Published:** 2017-12-11

**Authors:** María del Carmen Giménez-Espert, Vicente-Javier Prado-Gascó

**Affiliations:** 1 PhD, Professor, Facultad de Enfermería y Podología, Universidad de Valencia, Valencia, Spain.; 2 PhD, Professor, Facultad de Psicología, Universidad de Valencia, Valencia, Spain.

**Keywords:** Attitudes Towards Communication, Correlations, Emotional Intelligence, Empathy, Nursing, Sex

## Abstract

**Objectives:**

to analyze differences in the variables for the object of this study (attitude toward communication, emotional intelligence, and empathy) according to sex; verify correlations among variables between men and women and analyze regression models according to sex.

**Method:**

the ATC was used to measure attitudes toward communication; the Jefferson Scale of Empathy was used to measure empathy; and the Trait Meta Mood Scale 24 was used to measure emotional intelligence. The sample was composed of 450 nurses working in 7 hospitals located in Valencia, Spain. The t-test for independent samples was used to verify whether there were statistically significant differences, together with a prior application of the Levene test to assess the equality of variances. The correlations were analyzed using Person’s coefficient. Finally, the Beta coefficients of variables predicting ATC’s dimensions were verified using hierarchical multiple linear regression according to sex.

**Results:**

There are statistically significant differences based on sex for the variables, correlations and power of prediction.

**Conclusions:**

This study presents evidence on how the levels of variables (attitudes toward communication, EI, and empathy) vary among nurses according to sex, as well as the relationships established among such variables.

## Introduction

Interpersonal communication between nurses and patients is directly linked to care practices, as the basis of nursing care^1^ ^)^ and a central attribute of nursing care models. From this perspective, the communication skills of nurses are established as the central axis of quality of care^2^ and, therefore, of patient satisfaction^3^. In this sense, the literature suggests the existence of various factors modulating communication between nurses and patients, i.e., situational factors or extrinsic variables and dispositional factors or intrinsic factors. The first set of factors are related to the organizational conditions of health systems, which are often beyond the control of nurses, as these are both related to the physical environment and the localization of the various members of the multidisciplinary team^4^. Dispositional factors, or intrinsic variables, depend on the professional’s characteristics: age, sex, background, years of work, attitude, empathy, and emotional intelligence (EI)^5^, to mention a few. Communication is a dynamic, multidimensional and complex process^6^, thus, measuring it is difficult and requires multiple perspectives to be considered. Therefore, if communication with patients is considered a human behavior, its measurement should take into account attitudes. One’s attitude toward communication is probably one of the main determinants of nurse communication, since there is strong correlation between attitude and behavior^7^. Additionally, communication can be influenced by a nurse’s EI and empathy. EI enables nurses to properly regulate their emotions and those of others and is a very important requirement in key abilities, such as communication and empathy. Empathy is defined as one’s ability to read the emotions of others, putting oneself in another’s situation, understand one else’s thoughts and feelings^8^. Therefore, nurses with empathic abilities can understand a patient and establish a supporting relationship^9^. Considering the relationships existing among the variables under study, there are other sociodemographic variables, such as sex, that can affect relationships among them. Therefore, we need to describe the role the sex of professionals plays in these relationships. Women generally pay greater attention to their emotions^10^ and, for this reason, are more receptive to emotional support measures, while men are usually less attentive to their emotions^11^ ^-^ ^12^. This ability of women to pay greater attention to their emotions is an important resource for nurses, because it enables them to acquire a greater awareness of their own feelings and those of others, which is related to EI, and a greater understanding of a patient’s situation, which is related to empathy, so that the support provided by women is more significant^13^. These aspects are greatly important for nurses, because, even though men are increasingly choosing nursing as a profession, it remains a highly feminized profession^14^. Despite the importance of these aspects, there is a lack of studies assessing the moderating effect of sex on the relationships among such variables. In general, studies suggest that nurses lacking EI and empathy do not have the ability to communicate effectively, with patients nor the staff, leading to an unfavorable work climate and an increase in care delivery errors^15^. Patients are able to achieve a maximum level of wellbeing only when care is performed with effective communication skills^16^. Finally, EI is related to communication skills and a high EI is the proof that empathy and social skills are present^17^. For this reason, this study focuses on the role sex plays in some of the intrinsic variables, or dispositional factors, influencing communication, which are: attitude to communicate; empathy; and EI; as well as the relationships among such variables. Such understanding can improve working environments within the context of health to encourage and retain nurses who provide quality health care. 

## Method

This study’s population was composed of 450 nurses providing direct care to patients in 7 public hospitals in Valencia, Spain. Inclusion criteria were nurses actively working in the selected hospitals who had previously consented to participate in the study.

### Data collection procedure

After obtaining authorization from head nurses, the participants signed free and informed consent forms. The nurses filled out the questionnaires (taking approximately 35 minutes) and deposited them in the boxes located in the different services. After 2 weeks, reminders were sent by email and after 3-4 weeks, the questionnaires were collected. A total of 1,124 questionnaires were distributed, 460 of which returned, and ten discarded, as less than 60% of the instrument had been completed in those cases. Data were collected from June 2015 to March 2016.

### Data collection instruments

A self-administered instrument was used to collect data. The participants were supposed to respond to 3 instruments along with a form addressing sociodemographic data. The five-item Likert scale ranged from 1 to 5 (1=totally disagree and 5=totally agree). The instruments were the following:

Sociodemographic data: The participants provided information regarding their place of work; service; years of experience; sex; age; academic degree; and work situation. Finally, they were asked whether they had attended any specific training program addressing communication, empathy or EI.

Questionnaire addressing the attitudes of nurses toward communication (ATC). It is composed of 25 items comprising 3 dimensions: affective, cognitive, and conative, to assess their attitudes toward communication. In this study, the instrument presents appropriate psychometric properties, namely: Affective, Cronbach’s alpha=0.95; Conative, Cronbach’s alpha=0.92; and Cognitive, Cronbach’s alpha=0.85.

Jefferson Scale of Empathy for Nursing Students, adapted from the Jefferson Scale of Physician Empathy (JSPE)”^18^. It is composed of 19 items grouped into 3 factors and addresses empathy. It presents appropriate psychometric properties: Perspective Taken, Cronbach’s alpha=0.87; Compassionate Care, Cronbach’s alpha=0.78; and Putting oneself in another’s situation, Cronbach’s alpha=0.76.

Trait Meta-Mood Scale (TMMS24). It is a scale with 24 items grouped into 3 dimensions. The Spanish version was adapted by Fernández-Berrocal^19^ and is intended to assess EI. It also presents appropriate psychometric properties: Emotional attention, Cronbach’s alpha=0.80; Emotional clarity, Cronbach’s alpha=0.87 and Emotional Repair: Cronbach’s alpha=0.85.

### Data analysis

The differences in the variables that are the object of this study were first analyzed according to sex, then the correlations among the variables were calculated for men and women and, afterwards, regression models were also analyzed according to sex. To confirm whether there were statistically significant differences, a t-test for independent samples was used together with a prior application of the Levene test to prove equality of variances. The correlations were analyzed using Person’s coefficient. Finally, the Beta coefficients of the predictor variables of the ATC’s dimensions were determined according to sex using hierarchical multiple linear regression.

### Ethical aspects

The study was approved by the Institutional Review Board at the University of Valencia (H1432032268924) and at the selected hospitals. All the participants consented after having received clarification regarding the study’s objectives and procedures and were ensured of the confidentiality of information provided. 

## Results

### Participants’ sociodemographic characteristics

The participants’ ages ranged from 22 to 64 years old, with an average age of 44.13 years old (Standard Deviation=11.58). The distribution according to sex was: 75.6% (340) were women and 24.4% (110) were men. In regard to the participants’ academic education, 79.7% (321) had a bachelor’s degree, while 17.8% (72) had a Master’s degree, and 2.4% (10) had a doctoral degree. In regard to the participants’ occupational situations, 53.8% (239) had a stable work contract, 28.4% (126) were replacing other employees, while 17.8% (79) had a temporary contract. In terms of the years of experience, the participants presented from 5 months to 43 years and 3 months of experience, with an average of 18 years and 3 months (Mean=218.49 months; Standard Deviation=148.89 months), with a median of 5 years and 3 months. Finally, in regard to training programs addressing communication skills, empathy and/or emotional management, most participants, 50.1% (220 nurses), reported no training, while 38% (166 nurses) reported having received some training and 11.9% (52 nurses) reported having received considerable training.

### Comparison among the variables addressed by the ATC, JSE and TMMS24 according to sex

Statistically significant differences were found (p<0.05) only in regard to the dimensions of perspectives taking [t(390)=2.27; p=0.01; η^2^=0.20] and compassionate care [t(147.82)=-2.10; p=0.04; η^2^=0,10] of the JSE scale. The women (Mean=4.57; Standard Deviation=0.50) scored slighted higher than men (Mean=4.39; Standard Deviation=0.72) in the aspects related to perspectives taking. In the case of compassionate care, women (Mean=1.82; Standard Deviation=0.86) scored lower than men (Mean=2.03; Standard Deviation=0.98), in contrast with what happened in the other dimensions analyzed (Table 1).

**Table 1:** Dimensions of scales addressing Attitudes toward Communication, Jefferson Scale of Empathy, and Trait Meta-Mood Scale according to sex. Valencia, Spain, 2016

	**Dimensions**	**Women**		**Men**		Test t*	***p-value*** ^**†**^	**Effect** ^**‡**^
		**Mean**	**Standard Deviation**	**Mean**	**Standard Deviation**			
**Attitudes toward communication**	**Affective**	**1.55**	**0.87**	**1.65**	**0.91**	**0.92**	**0.36**	**§**
	**Conative**	**4.23**	**0.82**	**4.21**	**0.76**	**0.21**	**0.83**	**§**
	**Cognitive**	**4.54**	**0.80**	**4.40**	**0.88**	**1.48**	**0.14**	**§**
**Jefferson Scale of Empathy**	**Perspectives taking**	**4.57**	**0.50**	**4.39**	**0.72**	**2.27**	**0.01** ^**||**^	**0.20**
	**Compassionate care**	**1.82**	**0.86**	**2.03**	**0.98**	**-2.10**	**0.04** ^**¶**^	**0.10**
	**Putting oneself in another’s situation**	**1.99**	**1.03**	**2.18**	**1.02**	**-1.62**	**0.11**	**§**
**Trait Meta-MoodScale**	**Emotional Attention**	**3.58**	**0.77**	**3.57**	**0.77**	**0.16**	**0.87**	**§**
	**Emotional Clarity**	**3.83**	**0.69**	**3.83**	**0.69**	**-0.06**	**0.95**	**§**
	**Emotional Repair**	**3.81**	**0.77**	**3.84**	**0.76**	**-0.26**	**0.79**	**§**

*t-test; †p-value resulting from the Levene test; ‡Effect; §It was not calculated because there were no statistically significant differences; ||p≤0.01; p≤0.05.

Because the TMMS24 questionnaire has interpretative scores or scales, the percentages of women and men in the sample were verified according to the mean score, considering the scale of each of the TMMS24 dimensions. Most women were classified in the intermediary range of the scale for the 3 dimensions: 65.20% scored between 25 and 35 for emotional attention; 66.67% between 24 and 34 for emotional clarity; and 63.04% between 24 and 34 for emotional repair, which shows appropriate emotional attention, clarity and repair. Most men were also in the intermediary range of the scale for each of the TMMS24 dimensions: 70.21% scored between 22 and 32 in emotional attention; 64.52% between 26 and 35 in emotional clarity; and 64.21% scored between 24 and 35 in emotional repair, also indicating appropriate emotional attention, clarity and repair.

### Correlations among the ATC, JSE and TMMS24 according to sex

There is a statistically significant correlation among most of the dimensions of the 3 scales both for men and women. The highest correlations, for both groups, were found between dimensions in each scale separately. Note the correlations between pairs of factors on the ATC scale, both for men (r=-0.70 between affective and conative; r=-0.79 between affective and cognitive; and, r=0.81 between cognitive and conative) and women (r=-0.63 between affective and conative; r=-0.73 between affective and cognitive; and r=0.77 between cognitive and conative). The coefficients were higher among men than among women in most correlations. On the other hand, statistically significant correlations were not found among women between the ATC’s dimensions and emotional attention from the TMMS24 scale. In the case of men, no significant correlations were found between the affective and cognitive dimensions with emotional repair or between the conative variable and emotional attention (Table 2).

**Table 2:** Matrix of correlations using Person’s coefficient according to sex between the Attitudes toward Communication (ATC), Jefferson Scale of Empathy, and Trait Meta-Mood Scale. Valencia, Spain, 2016

	Affective ATC*		Conative ATC ATC*		Cognitive ATC*		**Perspective taking JSE** ^**†**^		**Compassionate care JSE** ^**†**^		**Putting oneself in another’s situation JSE** ^**†**^		**Emotional attention TMMS24** ^**‡**^		**Emotional clarity TMMS24** ^**‡**^	
	**M** ^**§**^	**W** ^**||**^	**M** ^**§**^	**W** ^**||**^	**M** ^**§**^	**W** ^**||**^	**M** ^**§**^	**W** ^**||**^	**M** ^**§**^	**W** ^**||**^	**M** ^**§**^	**W** ^**||**^	**M** ^**§**^	**W** ^**||**^	**M** ^**§**^	**W** ^**||**^
Affective ATC*																
Conative ATC*	**-0.70** ^**¶**^	**-0.63** ^**¶**^														
Cognitive ATC*	**-0.79** ^**¶**^	**-0.73** ^**¶**^	**0.81** ^**¶**^	**0.77** ^**¶**^												
**Perspectives taking JSE** ^**†**^	**-0.59** ^**¶**^	**-0.27** ^**¶**^	**0.63** ^**¶**^	**0.43** ^**¶**^	**0.52** ^**¶**^	**0.37** ^**¶**^										
**Compassionate care JSE** ^**†**^	**-0.59** ^**¶**^	**0.28** ^**¶**^	**0.63** ^**¶**^	**-0.19** ^**¶**^	**0.52** ^**¶**^	**-0.19** ^**¶**^	**1.00** ^**¶**^	**-0.45** ^**¶**^								
**Putting oneself in another’s situation JSE** ^**†**^	**0.32** ^**¶**^	**0.17** ^**¶**^	**-0.29** ^**¶**^	**-0.22** ^**¶**^	**-0.33** ^**¶**^	**-0.14** ^*^	**-0.36** ^**¶**^	**-0.28** ^**¶**^	**-0.36** ^**¶**^	**0.41** ^**¶**^						
**Emotional attention TMMS24** ^**‡**^	-0.22**	**-0.02**	**0.21**	**0.11**	**0.21** ^**^	**0.06**	**0.29** ^**¶**^	**0.23** ^**¶**^	**0.29** ^**¶**^	**-0.14** ^**^	**-0.14**	**-0.04**				
**Emotional clarity TMMS24** ^**‡**^	-0.25**	**-0.25** ^**¶**^	**0.32** ^**¶**^	**0.35** ^**¶**^	**0.31** ^**¶**^	**0.26** ^**¶**^	**0.30** ^**¶**^	**0.37** ^**¶**^	**0.30** ^**¶**^	**-0.21** ^**¶**^	**-0.14**	**-0.18** ^**¶**^	**0.41** ^**¶**^	**0.36** ^**¶**^		
**Emotional repair** **TMMS24** ^**‡**^	**-0.05**	**-0.17** ^**¶**^	**0.23** ^**^	**0.37** ^**¶**^	**0.00**	**0.26** ^**¶**^	**0.30** ^**¶**^	**0.41** ^**¶**^	**0.30** ^**¶**^	**-0.17** ^**¶**^	**-0.22** ^**^	**-0.14** ^**^	**0.26** ^**^	**0.21** ^**¶**^	**0.36** ^**¶**^	**0.57** ^**¶**^

*ATC - Attitude toward Communication Scale;†JSE - Jefferson Scale of Empathy; ‡TMMS24 - Trait Meta-Mood Scale; §M=Men; ||W=Women; ¶correlations are statistically significant at p<0.01; **correlations are statistically significant at p<0.05.

After presenting the correlations of variables according to sex, we confirmed the relationships among variables using multiple linear regression analyses, in which the predictor variables are the dimensions of the JSE and TMMS24 and the dependent variables or the outcomes are the dimensions presented by the ATC. 

### Hierarchical multiple linear regression

Finally, hierarchical multiple linear regression analysis was performed according to sex, using the ATC’s dimensions as criteria variables, while the JSE and TMMS24 were the predictor variables. All the dimensions addressed in the JSE were included in the first step, while the TMMS24 variables were included in the second step. In the first step, the JSE factors predicted 10% of the variance of the affective dimension, 16% of the cognitive (F=16.57), and 22% of the conative (F=23.03) dimensions in the women’s sample, while 36% of the variance of the affective (F=20.65), 31% of the cognitive (F=17.23), and 45% of the conative (F=30.58) dimensions were predicted in the male sample. The inclusion of the TMMS24’s dimensions as predictor variables in the second step did not significantly improve the explanation of the model since the increase in R^2^ ranged between 0.01 and 0.09 for men and between 0.02 and 0.05 for women. At this step, the emotional clarity dimension presented a statistically significant and positive beta coefficient for the affective dimension (β=-0.26; p<0.001) and also for the conative (β=0.14; p<0.05) dimension in the case of women. In the case of the perspectives taking dimension, the coefficients for women were: (β=-0.16; p<0.05) for the affective, (β=0.36; p<0.001) for the cognitive, and (β=0.37; p<0.001) for the conative dimensions. A significant and negative relationship was found between the conative dimension and the factor putting oneself in another’s situation (β=-0.12; p<0.05). In the case of men, the emotional repair dimension presented a significant and positive beta coefficient for the affective dimension (β=0.23; p<0.05), while a negative beta coefficient was found for the cognitive dimension (β=-0.29;p<0.01). Similarly, the coefficients found in the second step for the compassionate care dimension were: (β=-0.59; p<0.001) for the affective; (β=0.50; p<0.001) for the cognitive; and (β=0.62; p<0.001) for the conative dimensions. In regard to the total explained variance, in the case of women, it was 13% for the affective, 16% for the cognitive, and 23% for the conative dimensions, while for men, the variances were 36%, 36% and 42%, respectively. Thus, the JSE’s dimension perspectives taking and the TMMS24’s dimension emotional clarity contributed to predicting the ATC’s factors. These contributions were positive for the conative and cognitive factors and negative for the affective dimension, both for the variables perspective taking and emotional clarity. Also, the variable “putting oneself in another’s situation” contributed to predict the conative factor with a negative relationship. In the case of men, the variables that contributed to predicting the ATC’s factors were those concerning compassionate care, with a negative relationship in the affective dimension, and concerning the conative and cognitive dimensions, a positive relationship. Additionally, the variable emotional repair contributed to explaining the model, in the cognitive dimension with a negative relationship, and in the affective dimension with a positive relationship.

**Figure 1:**
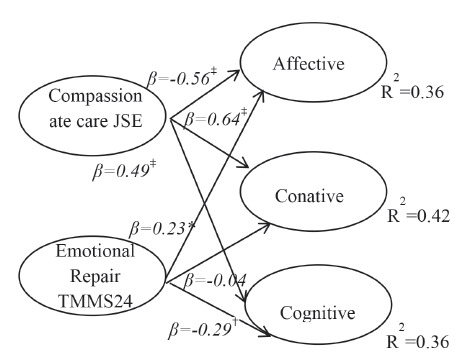
Model of relationships Men

Note: *p<0.05; †p<0.01; ‡p<0.001

**Figure 2:**
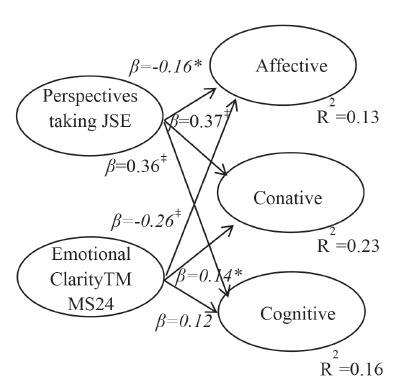
Model of relationships Women

Note: *p<0.05; †p<0.01; ‡p<0.001

## Discussion

This study’s results reveal statistically significant differences (p<0.05) only for the variable empathy in the JSE’s dimensions perspectives taking and compassionate care. Women showed a tendency to agreement slightly greater than men in the aspects related to perspective taking. In the case of compassionate care, women showed a slightly greater tendency than men for disagreement. The interpretation of results should consider that items related to compassionate care are negatively worded, thus, are inversely computed. The author of the original scale intended to avoid a tendency among respondents to always respond positively to questions, i.e., to avoid acquiescence bias. Such findings are in agreement with those reported by other studies, in which scores are slightly higher among women than among men^(13,20- 21)^. No significant differences were found between men and women in regard to the 3 dimensions of the TMMS24. Future studies should deepen investigation of these data as the literature^19^ confirms differences in terms of EI between men and women; i.e., women focus better and pay greater attention to their feelings. One potential explanation for a lack of difference between women and men in this specific case is that there are more women than men in the sample, a situation that is quite common in the context of nursing^14^ ^,^ ^22^. In the group of women, a statistically significant correlation was found among most dimensions of the scales under study, except between ATC’s dimensions and TMMS24’s emotional attention. In the group of men, all the variables were significantly correlated, except the affective and cognitive dimensions, with emotional repair. Additionally, no correlation was found between the conative behavioral variable and emotional attention. These findings suggest, in the case of women, that there is no relationship between emotional attention and communication that takes place daily. A possible explanation is that, in general, women are more emotional^23^ than men, so that it might be considered an innate characteristic that does not influence communication with patients. In the case of men, the importance they attach to communication is not related to feelings generated with emotional repair (ability to regulate emotions). These results suggest that, in general, men are systematic and pay less attention to their emotions, regulating feelings in a more rational manner^23^, as their attitude toward communication with patients, in the 3 dimensions, was not affected. Finally, considering the predictive models of the ATC based on the JSE and TMMS24, in general, EI and empathy present a greater predictive power among men than among women, considering that the percentage of explained variance among men ranged between 36% and 42%, while in the case of women, it ranged between 13% and 23%. Similarly, for the women, the JSE’s dimensions perspectives taking and putting oneself in another’s situation (only for the conative dimension) and the TMMS24’s emotional clarity, contributed to predicting the ATC’s factors, while for men, these predicted compassionate care and emotional repair. In this sense, in the case of women, perspective taking refers to the cognitive dimensions empathy and emotional clarity, and the ability to understand feelings. Thus, as one’s cognitive dimensions of empathy and EI increase, the easier it is to have a more favorable attitude toward communication with patients. Among men, the ability to properly regulate emotions and pay less attention to emotions may indicate a more favorable attitude toward communication with patients. Once again, the overall differences between men and women in regard to emotions^23^, in addition to differences assigned to the extrinsic characteristics of socialization and intrinsic characteristics of learned gender roles, may explain these results^24^.

One of the main limitations of this study is the sample. Non-probabilistic sampling procedures generally do not represent the population of nurses, and, even though a greater proportion of women was found, the results cannot be generalized. Another limitation is the use of self-reported instruments, a tool commonly used in investigations but that may introduce bias known as social desirability bias^25^. Therefore, other types of questionnaires and/or external objectives measures are recommended to detect differences between sexes. All these limitations will be taken into account in future investigations.

## Conclusion

This study presents evidence on how the levels of variables (attitudes toward communication, EI, and empathy) vary among nurses according to sex, as well as the relationships established among such variables. These findings enable the planning and assessment of nursing training programs to improve the levels of these variables among nurses. The assessment of these variables is essential given their repercussions for the quality of nursing care, thus for patient satisfaction.
